# The prognostic value of magnetic resonance imaging in moderate and severe traumatic brain injury: a systematic review and meta-analysis protocol

**DOI:** 10.1186/s13643-016-0184-x

**Published:** 2016-01-19

**Authors:** Hourmazd Haghbayan, Amélie Boutin, Mathieu Laflamme, François Lauzier, Michèle Shemilt, Lynne Moore, Ryan Zarychanski, Dean Fergusson, Alexis F. Turgeon

**Affiliations:** CHU de Québec—Université Laval Research Center, Population Health and Optimal Health Practices Research Unit (Trauma-Emergency-Critical Care Medicine), Université Laval, Québec, QC Canada; Department of Anesthesiology and Critical Care Medicine, Division of Critical Care Medicine, Université Laval, Québec, QC Canada; Department of Medicine, Université Laval, Québec, QC Canada; Department of Social and Preventive Medicine, Université Laval, Québec, QC Canada; Department of Internal Medicine, Section of Hematology/Medical Oncology and Critical Care Medicine, University of Manitoba, Winnipeg, MB Canada; CancerCare Manitoba, Department of Haematology and Medical Oncology, Winnipeg, MB Canada; Clinical Epidemiology Unit, Ottawa Hospital Research Institute, Ottawa, ON Canada

**Keywords:** Traumatic brain injury, Prognosis, Outcomes, Imaging, MRI

## Abstract

**Background:**

Traumatic brain injury (TBI) is a devastating condition with significant long-term mortality and morbidity. Despite current need for objective indicators to guide initial decision-making, few reliable acute phase prognostic factors have been identified. Early magnetic resonance imaging (MRI) has been investigated as a prognostic tool, but uncertainty remains in both its discriminative predictive value and which acute phase lesion patterns correlate with long-term outcome.

**Methods:**

We will conduct a systematic review of observational cohort studies and randomized controlled trials of adult moderate or severe TBI patients who underwent MRI in the acute phase after trauma. A high sensitivity search strategy will be employed in MEDLINE, EMBASE, BIOSIS, and Cochrane CENTRAL to identify citations. Two reviewers will independently screen all identified references for eligibility and extract data into a standardized form. Data will be collected on study design, baseline demographics, trauma characteristics, magnetic resonance (MR) technical specifications, lesion patterns, and outcomes as related to acute MRI imaging. If meta-analysis is possible, quantitative data for each outcome will be pooled per type of lesion pattern using random effects models and expressed as Mantel-Haenszel relative risks in order to determine the prognostic value of lesions detected on acute MRI and their strength as discriminatory predictors of long-term outcome. Statistical heterogeneity will be evaluated with the *I*^2^ statistics, and risk of bias and reporting quality will be assessed with standardized scales. Subgroup analyses are planned as a function of TBI severity, MRI-timing post-TBI, MRI field strength, MRI sequence, timing of outcome assessment, and risk of bias.

**Discussion:**

We expect significant clinical heterogeneity, as eligible studies will likely encompass different periods in evolving MRI technology in addition to significant variability of image sequence protocols and timing of acquisition between centers. Based on existing studies in TBI, we expect lesions detected in the brainstem to be of significant predictive value as MRI is particularly sensitive for imaging the brain’s posterior fossa. Our systematic review will allow clinicians to more accurately interpret MRI in the context of determining prognosis for moderate and severe TBI patients and inform researchers in this domain to improve the methodology of future studies.

**Systematic review registration:**

Prospero CRD42015017074

**Electronic supplementary material:**

The online version of this article (doi:10.1186/s13643-016-0184-x) contains supplementary material, which is available to authorized users.

## Background

Traumatic brain injury (TBI) is a significant global health problem, with the 1.7 million cases occurring annually representing upwards of $60 billion of direct and indirect health care costs in the USA alone [1, 2]. Moderate and severe TBI are most often life-threatening conditions requiring immediate intensive care. The determination of long-term neurological prognosis is thus of importance as it may inform patients or their representatives and better guide critical level of intervention decision-making [3]. Few reliable prognostic factors currently exist in this domain, with the large-scale IMPACT study identifying only age, pupillary reactivity, and the motor subscale of the Glasgow Coma Scale (GCS) as independent predictors of outcome [4, 5]. Recently, certain biomarkers [6] have also shown promise as outcome indicators; however, none of these factors are presently appropriate for clinical use.

Computed tomography (CT) currently plays a pivotal role in the immediate post-injury work-up where gross lesion characterization and indications for urgent surgical intervention must be rapidly established [7]. In the last four decades, magnetic resonance imaging (MRI) has emerged as a highly sensitive imaging tool in TBI. Its superiority compared to CT in detecting cerebral lesions in TBI, particularly non-hemorrhagic lesions and lesions localized to the posterior fossa, became evident just a few years following its clinical availability [8]. Visualization of the brainstem is especially crucial as a large volume of evidence from animal [9] and histological [10] studies have demonstrated that deeper, more caudally located lesions are correlated with greater severity of trauma. In continuity with this centripetal model [9] of brain injury, it has been proposed that such deeper lesions also have a greater significance on long-term outcome and may serve as prognostic indicators [11, 12]. Though several setbacks such as long imaging times and incompatibility of metallic objects have limited its use, advances in magnetic resonance technology are rapidly overcoming these obstacles giving MRI a growing role in the acute phase evaluation of TBI.

Over the last several decades, numerous studies have investigated the predictive value of MRI lesions. Owing at least in part to the diversity of approaches possible in interpreting cerebral lesions induced by TBI and correlating them to unfavorable long-term outcome, the results of such studies have been variable to date and at times contradictory. We seek to systematically identify all studies in this domain and to methodically synthesize their data studying MRI as a prognosticator in moderate and severe TBI. Our primary objective is to determine the prognostic value of MRI in TBI by identifying the lesion patterns that significantly correlate with mortality and neurological outcome. We also seek to investigate sources of possible heterogeneity and evaluate the methodological quality of the included studies.

## Methods

### Design

A team of experts including intensivists, internists, epidemiologists, and a biostatistician collaborated to develop the research question and study design of this systematic review, in accordance with the methodological guidelines delineated in the Cochrane Handbook for Systematic Reviews and Meta-Analyses [13]. This protocol was registered in PROSPERO (http://www.crd.york.ac.uk/PROSPERO/display_record.asp?ID=CRD42015017074) CRD42015017074. The final manuscript will be written in accordance with the Preferred Reporting Items for Systematic Reviews and Meta-Analyses (PRISMA) recommendations [14].

### Information sources and search strategy

MEDLINE, EMBASE, BIOSIS, and the Cochrane Central Register of Controlled Trials (CENTRAL) will be systematically searched from their inception, with an update planned before submission for publication. A three-pronged search strategy maximizing sensitivity has been developed to identify studies investigating MRI as a prognostic tool in TBI. Free text keywords, as well as Medical Subject Heading (MeSH) and Emtree terms, linked with the Boolean operator “OR” were used to design each prong of the search strategy, with the three prongs linked with the operator “AND.” All strategies will be reviewed by an information specialist (health care librarian) for robustness. After selection is complete, the reference lists of included studies will be reviewed to identify any additional eligible studies. An example of our search strategy is provided (Additional file 1).

### Eligibility criteria and study selection

The following inclusion criteria will be utilized to determine study eligibility: (1) cohort studies and randomized controlled trials (2) investigating the prognostic value of standard structural MRI (3) performed in the acute phase (≤28 days post-trauma) (4) of moderate or severe TBI (≥50 % with GCS ≤12) (5) in an adult population (≥80 % of patients aged ≥18 years old) (6) reporting at least one of our outcome measures of interest (mortality, Glasgow Outcome Scale (GOS), or extended Glasgow Outcome Scale (GOSe) as defined below). Studies with a significant population (>10 %) of penetrating TBI will be excluded. There will be no restriction based on publication date or language; translators will be consulted for articles published in languages other than English or French.

Two blinded reviewers will perform screening for study eligibility independently in a two-step process. Retrieved citations will initially be screened by title and abstract review for potential eligibility; retained studies will then be assessed by full-text analysis to confirm inclusion in the systematic review. A third reviewer will be consulted for arbitration in case of discordance. Reasons for exclusion at the full-text stage will be recorded and presented for transparency.

### Data collection

Two reviewers will independently extract data into a standardized data abstraction form, with a third to be consulted in cases of discordance. The following set of data will be extracted from each study: (1) study design, such as year, setting, study type, sample size, duration of follow-up, inclusion and exclusion criteria, sources of funding, and conflicts of interest; (2) patient characteristics, such as age, sex, comorbidities, and mechanism of injury; (3) therapeutic and supportive measures, such as use of mechanical ventilation, intracranial drains, and surgical intervention; (4) characteristics of the magnetic resonance imaging modality, such as time to scan, field strength, brand, sequences taken, and image plane; and (5) measures of outcome presented in relation to MRI image characteristics, such as lesion localization, lesion type, lesion size, and radiological scores, stratified by image sequence when possible. The initial data abstraction form will be piloted on five studies to ensure robustness, with subsequent modifications for thoroughness if necessary.

To avoid duplication, if the same study is published more than once, either the most complete article will be retained or all articles will be extracted and presented as a single study in analyses.

### Assessment of methodological quality

The methodological quality of any randomized controlled trials (RCTs) included in this systematic review will be evaluated with the Cochrane Collaboration’s risk of bias tool [13]. However, given that this is a prognostic systematic review and that we predict that the majority of the studies eligible for this review will have observational cohort designs, we modified the Quality in Prognostic Studies (QUIPS) tool [15] to develop a 26-item checklist appropriate for the evaluation of the risk of bias of such studies (see Additional file 2 for the complete tool). The QUIPS tool is a validated method for assessing the risk of bias in prognostic factor studies; we supplemented its list of searching questions with excerpts from the QUADAS-2 tool [16, 17] for additional rigor as we felt that neither framework alone encompassed all relevant questions regarding risk of bias. The STROBE statement’s [18] 22-item checklist will be used to evaluate the reporting quality of the included studies. By performing these assessments independently and in parallel, we seek to differentiate between methodological bias and omissions in reporting in the primary studies. Summaries of these evaluations will be presented in a graphical format to offer precise recommendations for future studies in this domain and, in the case of the risk of bias assessment, to also guide subgroup analysis. Both risk of bias and reporting quality evaluations will be performed independently by two reviewers.

### Quality of evidence

An adaptation of the GRADE framework for prognostic studies [19] will be employed to judge the quality of evidence for each outcome reported in this systematic review.

### Outcomes

Our primary outcomes will be mortality and unfavorable long-term Glasgow Outcome Scale (GOS or GOSe), defined as either a GOS of 1–3 or a GOSe of 1–4. Our secondary outcomes include duration of hospital stay, duration of ICU stay, any reported scales employed by the included studies to determine patient function (such as the Disability Rating Scale (DRS), the Craig Handicap Assessment and Rating Technique (CHART), and mini-mental state examination (MMSE)), and all other possible clinical end-point measures (such as coma duration, probability of readmission, and duration of rehabilitation).

### Statistical analysis and data synthesis

Data will be presented in a descriptive manner. Nominal variables and count data will be reported using proportions while continuous variables will be presented as either means with standard deviations or medians with ranges, depending on what is reported in the primary studies. If reported, effect measures will be presented in both their adjusted forms and unadjusted forms where possible. The number of studies reporting each type of lesion pattern in relation to outcome will be reported.

If meta-analysis is possible, random effects models will be employed. Dichotomous outcomes will be presented as risk ratios (RR) with accompanying 95 % confidence intervals (CIs) and forest plots, as generated with the Mantel-Haenszel method using Cochrane Review Manager version 5.2 (The Cochrane Collaboration, Copenhagen, Denmark, 2012). Data from the mortality and GOS will be presented at hospital discharge, 3, 6, and 12 months or beyond, according to the availability of the data.

Ordinal data will be presented in tabular format, with risks and relative risks for each study accompanied by 95 % CIs calculated using exact formulas. *P* values for global and trend tests will be computed for each study using SAS 9.3 (SAS Institute Inc., Cary, NC, USA, 2011). Ordinal data from radiological scores will also be dichotomized, when possible, according to the presence or absence of brainstem lesions and pooled using the same meta-analytical methods for dichotomic data as described above.

Heterogeneity will be evaluated by the *I*^2^ statistic and interpreted via the recommended standard categorization of negligible (<40 %), moderate (30–60 %), substantial (50–90 %), or considerable (75–100 %) [13]. Where permitted by the data available, sensitivity and subgroup analyses will be undertaken to explore sources of heterogeneity and test the robustness of the results. Such analyses will be performed in regard to minimal age of inclusion, severity of TBI, timing of MRI post-TBI, MRI field strength, MRI sequence, timing of outcome assessment, inter-rater reproducibility of image analysis, timing of outcome assessment, rehabilitation strategies, and study risk of bias. Visual analysis of funnel plots will be used to evaluate the presence and degree of publication bias.

## Discussion

Determination of long-term prognosis is an important step in the acute evaluation of moderate and severe TBI patients, particularly since a large proportion of such patients are young [1] with few or no comorbid conditions. Although a significant body of evidence has shown that MRI is superior to CT in detecting most types of traumatic parenchymal lesions [8, 20], only the latter is currently routine whereas use of the former remains sporadic in the acute phase. While the presence of several different lesion types, particularly those attaining the brainstem, has been correlated with severity of trauma [9, 10], the role of employing sensitive imagery such as MRI as an early prognosticator is not yet clear. In TBI patients, doubt remains concerning both the discriminatory ability of early MRI as well as which specific lesion patterns yield the highest prognostic information.

This project seeks to identify, class, and synthesize all existing original research with data relating lesions identified on acute MRI to clinical outcome in moderate and severe TBI patients. Our proposed systematic review of prognostic studies is based on well-recognized methodological [13, 16] and reporting [14] recommendations. It will determine the lesion patterns and radiological characteristics identifiable on acute MRI that correlate with the long-term outcome of patients having suffered moderate or severe TBI. By summating all existing evidence in the domain, the results of this systematic review will thus seek to conclusively inform clinicians and decision-makers on the significance, if any, of information provided by acute MRI in TBI and to explicitly establish its pertinence in the early management of moderate and severe TBI. Furthermore, by methodically classifying existing evidence and evaluating its risk of bias, our review seeks to also inform investigators of future studies in order to improve consistency in the approach to image interpretation and establish areas where further research is required.

Despite our intention to use a rigorous methodology and to employ a widely accepted statistical model for data analysis, we expect to likely encounter elevated clinical and statistical heterogeneity in the majority of our primary analyses. We anticipate this variability due to several factors, the most notable being that we expect that the majority of included studies will be of an observational cohort design. The pool of eligible studies will likely also encompass a significant variability in technical characteristics, due to both the evolution of MRI technology since its clinical introduction, as well as differences in sequence protocols and timing of imaging between study centers. Moreover, the method of image interpretation and lesion characterization is often variable, making it difficult to compare results across studies. To address such concerns regarding methodology, this systematic review will provide a global analysis of the quality of the evidence through the evaluation of both the risk of bias and the reporting quality of all the included studies via standardized assessment tools. The final resultant of this review will thus be both a systematic aggregate of the evidence that exists on the subject of prognostication in TBI via MRI as well as a critical appraisal of the methodology employed in this domain to ultimately also improve the quality of future studies. Our team plans to disseminate the results of the systematic review via presentation at research conferences and by publishing the results in a peer-reviewed journal.

## Additional files

Additional file 1:
**Example of Medline search strategy.** Description: In this file, we give an example of the Medline search strategy we will use for this study. (PDF 167 kb) Additional file 2:
**Evaluation of risk of bias.** Description: Adapted QUIPS tool with additions from QUADAS-2. (DOCX 35 kb)

## Abbreviations

CT: computed tomography
GCS: Glasgow Coma Scale
GOS: Glasgow Outcome Scale
GOSe: extended Glasgow Outcome Scale
MR: magnetic resonance
MRI: magnetic resonance imaging
RCT: randomized controlled trials
TBI: traumatic brain injury
