# Supplementary material for: The prognostic value of magnetic resonance imaging in moderate and severe traumatic brain injury: a systematic review and meta-analysis protocol
Source: Syst Rev. 2016 Jan 19;5:10. doi: 10.1186/s13643-016-0184-x (PMC4719205; doi:10.1186/s13643-016-0184-x)
Supplement: Additional file 1: — Example of Medline search strategy. Description: In this file, we give an example of the Medline search strategy we will use for this study. (PDF 167 kb) [file 13643_2016_184_MOESM1_ESM.pdf]

ADDITIONAL FILE 1: Example of Search Strategy (MEDLINE (Pubmed))

1. ((brain[TIAB] OR brains[TIAB] OR brainstem*[TIAB] OR head[TIAB] OR heads[TIAB] OR craniocerebral*[TIAB] OR intracrani*[TIAB] OR intra-crani*[TIAB] OR intercrani*[TIAB] OR inter-crani*[TIAB] OR cerebr*[TIAB] OR cerebel*[TIAB] OR forebrain*[TIAB]) AND (injury*[TIAB] OR injuries[TIAB] OR injured[TIAB] OR trauma[TIAB] OR traumas[TIAB] OR traumatic*[TIAB] OR traumato*[TIAB] OR damag*[TIAB])) OR TBI[TIAB] OR Craniocerebral Trauma[MeSH:NoExp] OR Brain Injuries[Mesh:NoExp] OR Brain Hemorrhage, Traumatic[Mesh] OR Diffuse Axonal Injury[Mesh:NoExp] OR Coma, Post-Head Injury[Mesh:NoExp] OR Head Injuries, Closed[Mesh:NoExp] OR Intracranial Hemorrhage, Traumatic[Mesh]

2. magnetic resonanc*[TIAB] OR “diffusion weighted”[TIAB] OR “diffusion tensor”[TIAB] OR MRI[TIAB] OR MR[TIAB] OR fMRI [TIAB] OR dMRI[TIAB] OR MRS[TIAB] OR MRA[TIAB] OR DTI[TIAB] OR DWI[TIAB] OR “T1-weighted”[TIAB] OR “T1 weighted”[TIAB] OR T1WI[TIAB] OR T1[TIAB] OR T1rho[TIAB] OR “T2-weighted”[TIAB] OR “T2 weighted”[TIAB] OR T2WI[TIAB] OR T2[TIAB] OR “T2*-weighted”[TIAB] OR “T2*WI”[TIAB] OR “T2*”[TIAB] OR “T2*-Gradient Echo”[TIAB] OR “T2*-GRE”[TIAB] OR “Fluid attenuated inversion recovery”[TIAB] OR FLAIR[TIAB] OR “Susceptibility weighted”[TIAB] OR SWI[TIAB] OR

“Magnetic Resonance Imaging”[MeSH:NoExp] OR “Diffusion Magnetic Resonance Imaging”[MeSH:Exp] OR “Echo-Planar Imaging”[MeSH:NoExp] OR “Magnetic Resonance Angiography”[MeSH:NoExp] OR

“Magnetic Resonance Imaging, Interventional”[MeSH]

3. Incidence[MeSH:NoExp] OR Mortality[MeSH Terms] OR Follow Up Studies[MeSH:NoExp] OR pognos*[Text Word] OR predict*[Text Word] OR course*[Text Word]

4. #**1** AND #**2** AND #**3**

5. animals[MeSH] NOT humans[MeSH]

6. #4 not #5
