# Supplementary material for: The prognostic value of magnetic resonance imaging in moderate and severe traumatic brain injury: a systematic review and meta-analysis protocol
Source: Syst Rev. 2016 Jan 19;5:10. doi: 10.1186/s13643-016-0184-x (PMC4719205; doi:10.1186/s13643-016-0184-x)
Supplement: Additional file 2: — Evaluation of risk of bias. Description: Adapted QUIPS tool with additions from QUADAS-2. (DOCX 35 kb) [file 13643_2016_184_MOESM2_ESM.docx]

**Additional file 2: Methodological Quality (Risk of Bias) Evaluation Tool for Studies of Prognostic Tests (Modified QUIPS tool with additions from QUADAS-2)**

| **Domain** | **Description** | **Judgment** | **Risk of bias** | **Applicability concerns** |
| --- | --- | --- | --- | --- |
| **01. Study Participation** | | | | |
| Was consecutive or appropriate random sampling used to enroll patients? (As opposed to voluntary sampling) | *Describe methods of patient selection*  *Describe included patients (previous testing, presentation, intended use of index test, and setting)* | □ Yes  □ No  □ Unclear | □ High  □ Low  □ Unclear |  |
| Was there adequate participation in the study by eligible individuals? | Low risk if:  - Majority (≥85%) of individuals meeting eligibility criteria participated in study | □ Yes  □ No  □ Unclear | □ High  □ Low  □ Unclear |  |
| Was a case-control design avoided? | Low risk if:  - Consecutive or random selection  *Careful in distinguishing bias vs. applicability concerns: judgement call | □ Yes  □ No  □ Unclear | □ High  □ Low  □ Unclear |  |
| Did the study avoid inappropriate exclusions? |  | □ Yes  □ No  □ Unclear | □ High  □ Low  □ Unclear |  |
| Are there concerns that the included patients do not match the review question? | - All adult (no paediatric) population  - Moderate or severe  - All pathologies are TBI  - Blunt head injury (no penetrative) | □ Adults  □ Moderate or severe  □ Blunt TBI |  |  |
| Overall | The study sample represents the population of interest on key characteristics, sufficient to limit potential bias of the observed relationship between the prognostic factor and outcome | □ True  □ False  □ Unclear | □ High  □ Low  □ Unclear | □ High  □ Low  □ Unclear |
| **02. Prognostic Factor Measurement** | | | | |
| *Blinding*  Were the prognostic marker results interpreted without knowledge of clinical data? | *Describe the prognostic marker and how it was conducted and interpreted* | □ Yes  □ No  □ Unclear | □ High  □ Low  □ Unclear |  |
| Was the method of prognostic factor measurement adequately valid to and reliable to limit misclassification bias? | Low risk if:  - Method of measurement recognized as valid or standard of practice in the domain  - Information on reliability/validity of method of measurement presented (ex. Cohen’s kappa, etc.)  - Diagnostic criteria presented | □ Yes  □ No  □ Unclear | □ High  □ Low  □ Unclear |  |
| *Inter-rater agreement*  Was inter-rater agreement evaluated via a statistical measure (ex. Cohen’s kappa)? |  | □ Yes  □ No  □ Unclear | □ High  □ Low  □ Unclear |  |
| If a threshold was used, was it specified *a priori*? [ □ Not applicable ] |  | □ Yes  □ No  □ Unclear | □ High  □ Low  □ Unclear |  |
| Were diagnostic criteria for prognostic factors well-defined? |  | □ Yes  □ No  □ Unclear | □ High  □ Low  □ Unclear |  |
| Are there concerns that the prognostic marker, its conduct, or its interpretation differ from the review question?  (including timing of assessment) | Low risk if:  - The method and setting of measurement of the prognostic factor is the same for all participants  Applicability concern if:  - Prognostic marker not associated to specific sequence (applicability issue; bias unlikely to be affected)  - Prognostic marker (MRI) measured over very long period | □ Specific sequence  □ Measured at specific time or over a short time window | □ High  □ Low  □ Unclear |  |
| Overall | The prognostic factor is adequately measured in study participants to sufficiently limit potential bias. | □ True  □ False  □ Unclear | □ High  □ Low  □ Unclear | □ High  □ Low  □ Unclear |
| **03. Outcome Measurement** | | | | |
| Was the outcome measurement adequate (evaluated reliably and validly)? | *Describe the outcome measurement and how it was conducted and interpreted* | □ Yes  □ No  □ Unclear | □ High  □ Low  □ Unclear |  |
| Were all patient outcomes evaluated identically? (method/setting standardized) | Low risk if:  - Data on reliability/validity  - Similar method of evaluation for all subjects  - Outcome evaluator blinded to patient history | □ Yes  □ No  □ Unclear | □ High  □ Low  □ Unclear |  |
| *Blinding*  Were the outcome results interpreted without knowledge of the results of the prognostic markers? |  | □ Yes  □ No  □ Unclear | □ High  □ Low  □ Unclear |  |
| Are there concerns that the prognostic marker, its conduct, or its interpretation differ from the review question?  (including timing of assessment) | Applicability concern if:  - Outcome measure reported as a binomial variable with a cut-off different from our review  - Outcome is measured or timing reported over very long intervals  - Minimum 1 measure of outcome at ≥6 months post-TBI | □ Same cut-off  □ Specific timing of assessment or short time window  □ Minimum of 1 measure at ≥6 months post-TBI |  |  |
| Overall | The outcome of interest is adequately measured in study participants to sufficiently limit potential bias. | □ True  □ False  □ Unclear | □ High  □ Low  □ Unclear | □ High  □ Low  □ Unclear |
| **04. Study Attrition** | | | | |
| Was the response rate (proportion of baseline sample completing the study and providing outcome data) adequate? Are there subjects not included in the analysis? | Low risk if:  - < 15% lost to follow-up  - Loss to follow-up but with multiple imputation method  - Intent-to-treat analysis | □ Yes  □ No  □ Unclear | □ High  □ Low  □ Unclear |  |
| Were all subjects included in the analysis? (Loss to follow-up? Withdrawal? Subjects not tested? Missing data? Etc.) |  | □ Yes  □ No  □ Unclear | □ High  □ Low  □ Unclear |  |
| Were attempts made to collect information on participants who dropped out or were lost to follow-up? | Reasonable attempts were made by investigators to acquire information on participants who did not complete the study and characterize them | □ Yes  □ No  □ Unclear | □ High  □ Low  □ Unclear |  |
| Are baseline characteristics and outcomes similar in patients who completed the study compared to those who did not? | Low risk if:  No important differences between participants having completed the study and those who did not | □ Yes  □ No  □ Unclear | □ High  □ Low  □ Unclear |  |
| Overall | The loss to follow-up or missing data affects a small proportion of the study participants and is not associated with key characteristics sufficient to limit potential bias to the observed relationship between the prognostic factor and outcome | □ True  □ False  □ Unclear | □ High  □ Low  □ Unclear | □ High  □ Low  □ Unclear |
| **05. Timing** | | | | |
| Was there an appropriate interval between the prognostic marker and the outcome measurement? (Biologically plausible, sufficient duration for outcome to occur) | *Describe the interval between prognostic markers and the outcome measurement* | □ Yes  □ No  □ Unclear | □ High  □ Low  □ Unclear |  |
|  | Low risk if:  - Mortality or GOS measured at hospital discharge or later |  |  |  |
| Overall | The interval of time between measurements of the prognostic factor and outcome is adequate to respond to the study hypothesis | □ True  □ False  □ Unclear | □ High  □ Low  □ Unclear | □ High  □ Low  □ Unclear |
| **06. Study Confounding** | | | | |
| Did the study adequately control for potential confounders?  Are important potential confounders accounted for in the analysis? | *Describe any method used to control for potential confounding* | □ Yes  □ No  □ Unclear | □ High  □ Low  □ Unclear |  |
|  | Low if:  - Adjustment for: age, motor subscale of the GCS, and pupillary reactivity  High if:  - No adjusted measure reported  - Inadequate adjustment (important variables not taken into account, improper statistical method used (ex. forward model)) |  |  |  |
| Are the measurements of all important confounders adequately valid and reliable? | Method of measurement recognized as valid or standard of practice in the domain | □ Yes  □ No  □ Unclear | □ High  □ Low  □ Unclear |  |
|  | The method and setting of confounding measurement are the same for all study participants |  |  |  |
| Overall | Important potential confounders are appropriately accounted for, limiting potential bias with respect to the relationship between the prognostic factor and outcome. | □ True  □ False  □ Unclear | □ High  □ Low  □ Unclear | □ High  □ Low  □ Unclear |
| **07. Statistical Analysis** | | | | |
| Are the statistical methods employed in the study adequate? | The statistical tests/methods are appropriate for the type of data analyzed and are adequate for testing the study hypothesis | □ Yes  □ No  □ Unclear | □ High  □ Low  □ Unclear |  |
| Is the model development strategy adequate (if applicable)? | The strategy for model building is appropriate and is based on a conceptual framework or model | □ Yes  □ No  □ Unclear | □ High  □ Low  □ Unclear |  |
|  | The selected statistical model is adequate for the design of the study. |  |  |  |
| Were all statistical analyses pre-specified? | Low if:  - No post-hoc data analysis  Reviewer’s judgement if:  - Post-hoc analysis clearly identified as such and adequately justified in discussion | □ Yes  □ No  □ Unclear | □ High  □ Low  □ Unclear |  |
| Overall | The statistical analysis is appropriate for the design and hypothesis of the study, limiting potential for invalid or spurious results. | □ True  □ False  □ Unclear | □ High  □ Low  □ Unclear | □ High  □ Low  □ Unclear |

Modified from:

- Hayden JA, van der Windt DA, Cartwright JL, Cote P, Bombardier C. Assessing bias in studies of prognostic factors. *Annals of Internal Medicine*. 2013;158(4):280-6.
- Whiting PF, Rutjes AW, Westwood ME, Mallett S, Deeks JJ, Reitsma JB et al. QUADAS-2: a revised tool for the quality assessment of diagnostic accuracy studies. *Annals of Internal Medicine*. 2011;155(8):529-36
